# Clinical benefits of central pancreatectomy for a patient with pancreatic schwannoma and diabetes

**DOI:** 10.1186/s12957-024-03646-5

**Published:** 2025-01-03

**Authors:** Long Cheng Zhao, Zi Ye Li, Fan Wu, Yue Hu, Bai Lin Wang

**Affiliations:** 1 Department of Hepatobiliary Surgery, Guangzhou Red Cross Hospital of Jinan University, Tongfu Roud 396, Guangzhou, 510220 Guangdong China; 2 Department of Pathology, Guangzhou Red Cross Hospital of Jinan University, Guangzhou, China

**Keywords:** Pancreatic neoplasms, Schwannoma, Central pancreatectomy

## Abstract

Schwannomas are tumors that originate from the glial cells of the nervous system and can occur on myelinated nerve fibers throughout the body, especially in the craniofacial region. However, pancreatic schwannomas are extremely rare. We report a case of a pancreatic schwannoma that was difficult to differentiate from other pancreatic tumors preoperatively. A 44-year-old female patient was found to have a pancreatic mass on Computed Tomography imaging and the preoperative diagnosis was a pancreatic solid pseudopapillary neoplasm. Meanwhile, the patient had type 2 diabetes and the blood glucose was controlled at 8-15mmol/L by taking oral antidiabetic drugs. During exploratory laparotomy, an 8 cm × 7 cm × 4 cm mass was discovered in the middle part of the pancreas. Considering the preoperative diabetes, the patient underwent a central pancreatectomy (CP) and Roux-en-Y pancreaticojejunostomy. Postoperative histopathological examination confirmed the diagnosis of a pancreatic epitheloid schwannoma. After surgery, the patient developed Grade B pancreatic fistula, which disappeared after treatment. At the same time, the patient’s blood glucose remained basically stable by insulin therapy, which was adjusted to oral antidiabetic medications in about 40 days after surgery. At a 32-month follow-up after discharge, no tumor recurrence was observed, and the patient’s blood glucose was controlled below 11.1mmol/L with only oral antidiabetic drugs. The radiological diagnosis of pancreatic schwannomas lacks specific features, and diagnosis primarily relies on histopathological examination and immunohistochemical testing. Although pancreatic schwannomas are extremely rare, they must be differentiated from other solid or cystic pancreatic lesions. For patients with pancreatic schwannoma and diabetes, CP may represent a favorable surgical option.

**Supplementary Information:**

The online version contains supplementary material available at 10.1186/s12957-024-03646-5.

## Introduction

Schwannomas are tumors originating from the Schwann cells of peripheral nerves. Approximately 25–45% of schwannomas occur in the head and neck region [1], followed by the limbs [2]. Schwannoma in the pancreas is extremely rare. Pancreatic schwannomas are usually solid or cystic benign tumors, though some may have a tendency for malignant transformation [3], and their pathogenesis remains unclear. It primarily affects individuals between the ages of 20 and 50, with no gender preference. Most patients present with gastrointestinal symptoms, such as nausea, vomiting, and indigestion, although some cases are asymptomatic. Currently, the treatment for pancreatic schwannomas primarily involves surgical resection. Pancreaticoduodenectomy (PD) and distal pancreatectomy (DP) are the main surgical approaches reported in most cases, with only one report detailing a case where central pancreatectomy (CP) was performed [4]. In this article, we present a report on a 44-year-old female patient with pancreatic schwannoma and diabetes who underwent CP, and conduct a review of the relevant literature.

**Fig. 1:**
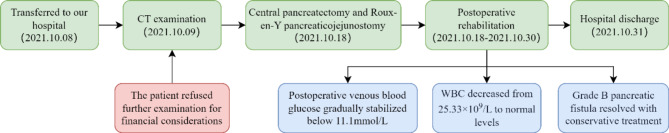
The timeline of key events during patient care

**Fig. 2:**
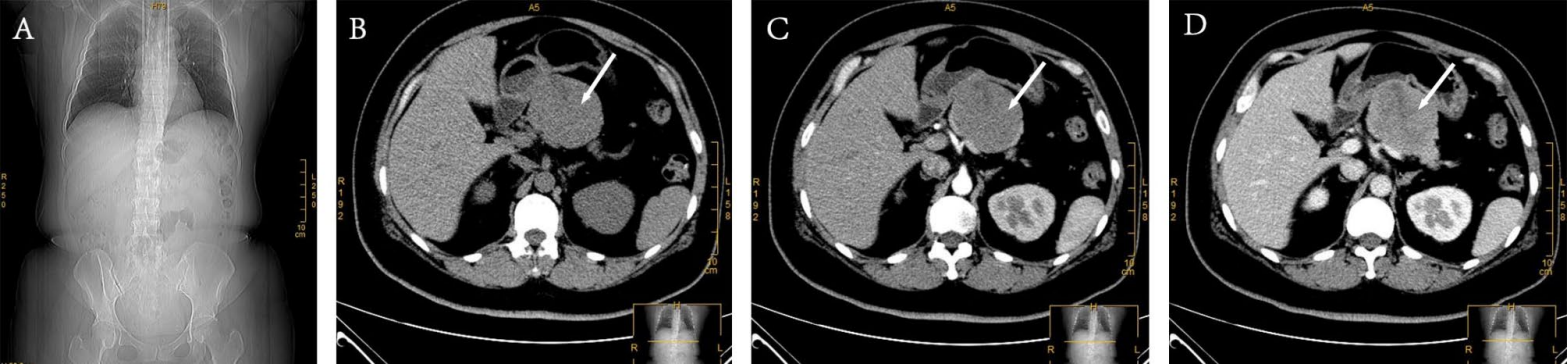
Preoperative abdominal CT. **A**: Abdominal x-ray plain films; **B**: Non-contrast enhanced CT; **C**: Arterial phase of contrast-enhanced CT; **D**: Venous phase of contrast-enhanced CT; Note: The tumor indicated by the arrow in the figure is a pancreatic schwannoma. It is a low-density solid mass under non-contrast enhanced CT, and heterogeneous enhancement can be seen under contrast-enhanced CT

In 2021, a 44-year-old female presented to a local hospital with upper abdominal discomfort. Abdominal Computed Tomography (CT) revealed a pancreatic mass, and she was subsequently transferred to our hospital for further treatment (Fig. 1). Physical examination showed a deep mass in the upper abdomen, approximately 7 cm × 7 cm in size, with a hard consistency and poor mobility. No other significant abnormalities were noted on the rest of the examination. The patient had a 2-year history of type 2 diabetes with poor medication control. Tumor markers, including CEA, CA19-9, and CA72-4, were within normal limits, but neuron-specific enolase (NSE) was elevated. Insulin: 36.57 mIU/L, C-peptide: 1.9 nmol/L, albumin: 37.6 g/L and LDH: 201 U/L. Abdominal CT revealed a 64 mm × 54 mm mass in the body of the pancreas, with clear borders and no enhancement on the slice, and mild dilation of the main pancreatic duct was observed, but no evidence of metastasis was found (Fig. 2). Due to the patient’s financial constraints, she refused a magnetic resonance imaging (MRI) scan, which hindered the accuracy of our diagnosis. MRI, with its ability to assess tumor characteristics through various sequences such as T1 and T2, can more accurately display the tumor’s morphology and its relationship with surrounding tissues, which is extremely helpful for diagnosing solid tumors. Clinically, pancreatic cystic tumors are more common than pancreatic schwannomas, and the CT features of pancreatic solid pseudopapillary neoplasms (pSPN) can closely resemble those of pancreatic schwannomas. Based on the patient’s symptoms and laboratory results, our preliminary diagnosis was pSPN. The Royal Marsden Hospital score indicated a low-risk group, suggesting a relatively favorable prognosis [5].

**Fig. 3:**
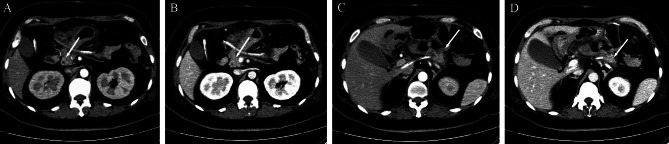
Postoperative CT images after Roux-en-Y Pancreaticojejunostomy. **A**: Arterial phase of contrast-enhanced CT; **B**: Venous phase of contrast-enhanced CT; **C**: Arterial phase of contrast-enhanced CT; **D**: Venous phase of contrast-enhanced CT; Note: The arrows in pictures **A** and **B** indicate the residual pancreatic head; The arrow in picture **C** indicates the location of the anastomosis of pancreaticojejunostomy; The arrow in picture **D** indicates the residual pancreatic tail

**Fig. 4:**
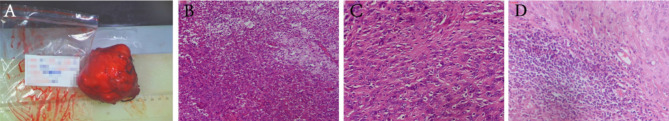
Pathological photograph. **A**: Resected pancreatic schwannoma; **B**: H&E ×100, a large amount of foamy histiocytes were deposited in the interstitium; **C**: H&E ×200, the epithelioid tumor cells were arranged in strips and sheets, and the stroma was collagenous; **D**: H&E ×200, lymphocyte aggregation at the edge of the tumor

**Fig. 5:**
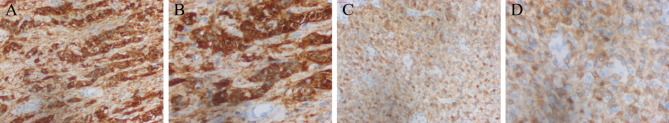
Immunohistochemical staining picture. **A**: S100 (+) ×200; **B**: S100 (+) ×400; **C**: NSE (+) ×200; **D**: NSE (+) ×400

After obtaining informed consent from the patient and her family, our treatment team performed an exploratory laparotomy. During surgery, a mass approximately 8 cm × 7 cm × 4 cm was palpated in the body of the pancreas. Therefore, the patient underwent CP and Roux-en-Y pancreaticojejunostomy. The postoperative CT images of the patient are shown in Fig. 3, the direction indicated by the arrows in pictures A and B shows the pancreatic remnant, while the arrows in pictures C and D indicate the location of Roux-en-Y pancreaticojejunostomy, with the pancreatic stent clearly visible at the central part of the pancreas. Frozen section analysis was performed on the mass that was completely resected. The frozen section labeled “pancreatic mass” revealed tumor cells that were polygonal or round in shape, uniform in morphology, and arranged in cords or blocks. Foam-like stromal cells were observed. We suspected that the mass was a pSPN, which needs to be differentiated from pancreatic neuroendocrine neoplasm. The paraffin section showed that the tumor cells had round or polygonal nuclei, with fine granular chromatin. Some nuclei contained visible nucleoli, and the cellular boundaries were not well defined. The cells were arranged in a palisading pattern in some areas, and the other were arranged in a rope-like or fascicular pattern, or in a pseudo-glandular or sheet-like arrangement (Fig. 4). Immunohistochemical results (Fig. 5): S100 (+), P53 (+), CK5/6 (-), CD56 (+), CD68 (+), Ki − 67 hot zone (< 5% +), NSE (+). The diagnosis was pancreatic schwannoma.

After surgery, the patient developed abdominal pain and fever. Amylase and lipase levels in the abdominal drain fluid were elevated, indicating a Grade B pancreatic fistula. The patient was treated symptomatically with fasting, nutritional support, antibiotics, and gastric lavage. After these treatments, her symptoms resolved, and the amylase levels in the drain fluid returned to normal. Preoperatively, her fasting venous blood glucose was approximately 8–15 mmol/L, controlled by oral medications, but with poor efficacy. Postoperatively, her fasting venous blood glucose fluctuated between 12 and 20 mmol/L with insulin therapy. After her feeding, subcutaneous insulin injections were used to maintain blood glucose levels below 11.1 mmol/L. About 40 days after surgery, her treatment was adjusted to oral hypoglycemic medications, and her venous blood glucose was stabilized at around 10 mmol/L. At a 32-month follow-up after discharge, no tumor recurrence was observed, and the patient’s blood glucose was controlled below 11.1mmol/L with only oral antidiabetic drugs. The patient fully understood the purpose of this case report and its contents, and she signed an informed consent form allowing the publication of her relevant medical information.

## Discussion

Schwannomas are tumors originating from Schwann cells, which surround the myelinated nerve fibers. Schwannomas are generally benign, with approximately 10–15% undergoing malignant transformation [6]. These tumors are most commonly found in the limbs, neck, mediastinum, retroperitoneum, and posterior nerve roots of the spinal cord [7]. The majority of patients present initially with a painless mass, and other signs and symptoms vary depending on the tumor’s anatomical location [8]. Zhang included 75 reported cases of pancreatic schwannomas, with abdominal pain being the most common symptom (44%), followed by asymptomatic patients (31%), and other symptoms include weight loss, mass, and jaundice [9]. Pancreatic schwannomas are extremely rare [10], and their growth pattern is similar to that of schwannomas found in other parts of the body. However, pancreatic schwannomas typically present with nonspecific abdominal pain [11]. The most common location for pancreatic schwannomas is the head of the pancreas, followed by the body, tail, and uncinate process [12]. A literature search was conducted in September 2024. The MeSH term “pancreatic schwannoma” was used in searches on both PubMed and China National Knowledge Infrastructure (CNKI). The PubMed search for the past decade yielded 38 articles describing 41 detailed cases of pancreatic schwannoma in the English literature. The CNKI search for the past decade identified 4 articles describing 4 detailed cases of pancreatic schwannoma in the Chinese literature (Detailed documents are provided in the supplementary materials). We analyzed and summarized the 45 cases of pancreatic schwannoma identified from the searches, with clinical and pathological data summarized in Table 1.

**Table 1:** Summary of clinicopathological data from all 45 cases of pancreatic schwannoma reported in the recent 10 years

	*N* (%) or Mean ± SD
Age (year) (*n* = 45)	
≤ 30	4
30–60	22
≥ 60	19
	55.43 ± 14.839
Sex (*n* = 45)	
Male	15
Female	30
Male: Female	1:2
Symptoms (*n* = 41)	
Abdominal pain	20(48.78%)
Abdominal bloating	2 (4.88%)
Diarrhea	1(2.44%)
Nausea/ Vomiting	3(7.32%)
Indigestion	3(7.32%)
Weight loss	4(9.76%)
Jaundice	2(4.88%)
No symptoms	17(41.46%)
Tumor location (*n* = 45)	
Head	19(42.22%)
Head + body	6(13.33%)
Body	11(24.44%)
Body + Tail	1(2.22%)
Tail	8(17.78%)
Nature of tumor on imaging (*n* = 44)	
Soild	28(63.64%)
Cystic	9(20.45%)
Soild + Cystic	7(15.91%)
Preoperative diagnosis (*n* = 36)	
Pancreatic Schwannoma	16(44.44%)
Pancreatic cystadenoma	8(22.22%)
Pancreatic solid pseudopapillary neoplasm	8(22.22%)
Neuroendocrine neoplasm	1(2.78%)
Acinic cell carcinoma	1(2.78%)
Pancreatic cancer	2(5.56%)
Accuracy	35.60%
Surgical methods (*n* = 40)	
Enucleation of tumor	10(25.00%)
Pancreaticoduodenectomy	9(22.50%)
Distal pancreatectomy	11(27.50%)
Central pancreatectomy	2(5.00%)
Conservative treatment	8(20.00%)

Note: Because some patients come in with multiple symptoms, the percentage in the symptoms column will be greater than 100%

Due to the lack of specific diagnostic methods, preoperative diagnosis of pancreatic schwannoma is challenging. In the absence of pathological results, imaging is often a key tool for preoperative diagnosis. On CT, pancreatic schwannomas typically present as well-defined, round or oval masses with clear borders, marked cystic degeneration, and punctate calcifications. CT contrast enhancement shows localized cystic changes within the tumor, with areas of low density and no enhancement [13]. Malignant transformation of pancreatic schwannomas is characterized by rapid growth, infiltration of surrounding tissues, and the presence of irregularly shaped, solid, heterogeneous masses, with possible lymph node metastasis [14]. Additionally, the tumor may show the formation of vascular thrombosis. On MRI, a well-defined pancreatic mass appears as heterogeneous high signal intensity on T2-weighted images, with distinct low signal intensity on T1-weighted images, and high signal intensity on diffusion-weighted imaging. The mass shows mild enhancement in the arterial phase, with further enhancement in the portal venous and delayed phases. These imaging features suggest a possible diagnosis of pancreatic schwannoma [15]. The diagnosis of schwannoma requires differentiation from other pancreatic tumors, such as pancreatic cystic tumors, pancreatic neuroendocrine neoplasms, pancreatic solid pseudopapillary neoplasms (pSPN), and pancreatic cancer. Pancreatic cystic tumors primarily present as cystic lesions on imaging, characterized by fluid-filled dark areas, often with multilocular structures and minimal solid components, which differ significantly from pancreatic schwannomas. Pancreatic neuroendocrine neoplasms share both cystic and solid components, similar to schwannomas, but neuroendocrine neoplasms tend to exhibit a dense vascular pattern, leading to homogeneous enhancement on contrast-enhanced CT [16], which is not consistent with the imaging features of pancreatic schwannomas. pSPN are also mixed solid-cystic masses, making them difficult to distinguish from pancreatic schwannomas. Moreover, pSPN can also present as cystic masses or calcified cystic tumors [16, 17]. Although pancreatic schwannoma and pSPN have similar imaging findings, pSPN does not express NSE, whereas pancreatic schwannoma does. Therefore, these two diseases can be differentiated through a combination of imaging studies and laboratory examinations. Early pancreatic cancer can present as a solitary solid mass similar to pancreatic schwannoma. However, pancreatic cancer has distinct features, such as elevated CA-199 levels, significant enhancement on contrast-enhanced CT and clear signs of tissue invasion, which help differentiate it from pancreatic schwannomas. Compared to CT, PET/CT is more sensitive for the diagnosis of pancreatic cancer [18]. Therefore, in our data, the misdiagnosis rate for pancreatic cancer is relatively low. Since the first case of endoscopic ultrasound-guided fine-needle aspiration (EUS-FNA) was performed in 1997 [19], EUS-FNA has been very helpful for the preoperative diagnosis of pancreatic schwannoma [20–24]. With the development of technology, the sensitivity of EUS-FNA for determining the nature of a tumor can exceed 90%, with a specificity of over 97% [25, 26]. This technique plays a crucial role in formulating precise treatment plans, not only optimizing medical decisions but also significantly improving treatment outcomes and prognosis for patients.

Currently, the diagnosis of pancreatic schwannoma mainly relies on histopathology and immunohistochemical staining. Pancreatic schwannomas are uniform, yellow-brown nodules with clear boundaries and an intact capsule observed macroscopically [27–29]. Microscopically, they typically exhibit two types of tissue structures: Antoni A and Antoni B. The Antoni A area is characterized by a rich presence of spindle-shaped cells, usually arranged in a palisade pattern or forming Verocay bodies (Fig. 3C). Tumor cells in the Antoni A area have very few mitotic figures, typically less than 5 mitotic figures per 10 high-power fields [30, 31]. In contrast, the Antoni B area has fewer tumor cells, which are arranged in a sparse network-like structure. There is a large amount of fluid and mucinous matrix within and between cells, forming cystic structures, typically exhibiting degenerative changes such as myxoid changes, cyst formation, stromal hemorrhage, and calcification [32]. On CT, Antoni A-type pancreatic schwannomas appear as low-density solid masses with an uneven enhancement pattern, occasionally with multiple septal enhancements. Antoni B-type pancreatic schwannomas tend to appear as homogeneous cystic or multiple masses [33]. The more vascularized Antoni A areas typically show enhancement, while Antoni B areas show no enhancement [34]. Almost all benign schwannomas contain abundant S100 (+) cells, while only about 50% of malignant schwannomas show S100 (-), suggesting that S100 can be used as an initial marker to differentiate between benign and malignant schwannomas [30, 35–38]. NSE is a glycolytic enzyme isozyme primarily found in the cytoplasm of central and peripheral neurons, as well as neuroendocrine cells, and is an important marker for diagnosing various neuroendocrine neoplasm [39, 40]. Through literature review, we found that pancreatic tissue-derived tumors rarely express this enzyme [41]. Therefore, the strong positive staining for S100 and NSE in this case provides solid evidence for the diagnosis of pancreatic schwannoma.

Most schwannomas grow slowly, with an average growth rate of 1.2 mm per year [42]. Small schwannomas can be monitored periodically [43]. However, for symptomatic schwannomas, surgical treatment is necessary. Regarding surgical options for pancreatic schwannoma, in cases with a confirmed diagnosis, complete resection can achieve the therapeutic goal. However, if the preoperative diagnosis is unclear, the tumor should be completely resected during surgery, and frozen section pathology should be performed to determine the extent of resection. In a previous review of 65 cases of pancreatic schwannomas, Fukuhara et al. found that schwannomas most commonly occur in the head of the pancreas (40%), followed by the body (23.1%), tail (10.8%), and uncinate process (10.8%). The most common treatment approach is pancreaticoduodenectomy (34%), followed by distal pancreatectomy (25%) and enucleation (14%). The pancreas is a key organ responsible for secreting various hormones and digestive enzymes. Insulin and glucagon are secreted by the β-cells and α-cells of the pancreas, respectively, and play a central role in glucose metabolism [44]. Pancreatic resection can be categorized into two main types: partial and total. Total pancreatic resection results in complete loss of both endocrine and exocrine functions of the pancreas, leading to difficulty in achieving glucose control [45]. In contrast, partial pancreatic resection preserves both the endocrine and exocrine functions of the pancreas, making it easier to manage blood glucose levels compared to total pancreatic resection. Partial pancreatic resection can be further subdivided into pancreaticoduodenectomy (PD), distal pancreatectomy (DP), and central pancreatectomy (CP). After PD, about 50% of the pancreatic tissue remains, which leads to a reduction in the secretion of insulin and glucagon [46]. For patients with preexisting diabetes, this operation may worsen their condition. Additionally, PD significantly alters the digestive system and reduces exocrine function [47], making it unacceptable for patients with non-malignant tumors who do not require radical surgery [48]. After DP, approximately 30-40% of the pancreatic tissue remains [49, 50]. Compared to PD, DP has a relatively smaller impact on the structure of the digestive system. However, this operation inevitably involves the removal of a considerable amount of healthy pancreatic tissue, which can significantly affect the postoperative recovery of pancreatic function [51]. In contrast, CP preserves more pancreatic tissue (and sometimes the spleen), which greatly facilitates the recovery of pancreatic function post-surgery. Studies have shown that the incidence of new-onset diabetes after CP is lower than after PD and DP [51], suggesting that CP has a lesser impact on pancreatic function and a better blood glucose control for diabetic patients. However, CP also has certain drawbacks. Due to the necessity of carefully managing both ends of the pancreatic remnant, CP requires longer operating times and is associated with a higher incidence of pancreatic fistula compared to PD and DP. A meta-analysis by Bi et al. comparing the advantages and disadvantages of DP and CP supports this conclusion. The surgical time in the DP group was significantly shorter than CP group, but intraoperative blood loss was higher in the DP group. Regarding postoperative complications, the incidence of pancreatic fistula in the CP group (36.9%) was significantly higher than DP group (20.2%). The incidence of severe postoperative complications (Clavien-Dindo grade III or higher) in the CP group (21.8%) was also higher than DP group (12.8%). However, the incidence of endocrine insufficiency after surgery in the CP group (6.7%) was much lower than DP group (20.6%), and the incidence of new-onset or worsened diabetes in the CP group was also lower than DP group [52]. On the other hand, another article indicated no significant difference in the probability of pancreatic fistula between the CP and DP groups [53]. This discrepancy may be attributed to the surgeon’s technical skills, suggesting that CP can minimize its drawbacks and effectively prevent postoperative metabolic disorders through precise technique and enhanced postoperative care, ultimately ensuring a higher quality of life for patients after surgery. Additionally, after comparing 34 patients in the CP group and 262 patients in the DP group, Chen YW et al. found that no new-onset or worsening diabetes occurred in the CP group, while 40 patients in the DP group developed endocrine insufficiency after surgery (*P* < 0.05), and the incidence of exocrine insufficiency was significantly higher in the DP group [54]. Some studies have pointed out that poor blood glucose control increases the risk of surgical site infections [55]. Therefore, CP can preserve both endocrine and exocrine pancreatic functions postoperatively, reducing the incidence of new-onset or worsening diabetes [56], which offers long-term benefits for the patients. In this case, the patient’s diabetes remained stable after surgery, with oral medication treatment, demonstrating the therapeutic value of CP for patients with pancreatic schwannomas and diabetes.

In conclusion, pancreatic schwannoma is a rare disease that presents unique challenges in both diagnosis and treatment. Due to the lack of specific clinical symptoms and typical imaging features, the preoperative misdiagnosis rate remains high, making it a significant challenge to improve diagnostic accuracy. However, once diagnosed, surgical treatment typically yields favorable outcomes and prognosis. In this case, we chose CP and achieved significant therapeutic success. Our treatment experience, combined with findings from previous literature, suggests that CP may be a more ideal surgical approach for patients with pancreatic schwannoma and diabetes.

## Electronic supplementary material

Below is the link to the electronic supplementary material.

Supplementary Material 1

Supplementary Material 2

## Data Availability

No datasets were generated or analysed during the current study.

## References

[1] He X, Wang Y. Neurilemmoma of the Nasal Cavity and Paranasal Sinuses. Ent-Ear Nose Throat. 2021;102(7):NP364–NP368. 10.1177/01455613211007947. 10.1177/01455613211007947 33951978

[2] Pilavaki M, Chourmouzi D, Kiziridou A, et al. Imaging of peripheral nerve sheath tumors with pathologic correlation: pictorial review. Eur J Radiol. 2004;52(3):229–39. 10.1016/j.ejrad.2003.12.001. 10.1016/j.ejrad.2003.12.001 15544900

[3] Hosmann A, Kamdar V, Misra BK. Malignant transformation of vestibular schwannoma following radiosurgery-a case report and review of the literature. Acta Neurochir. 2024;166(1):52. 10.1007/s00701-024-05921-6. 10.1007/s00701-024-05921-6 38289497

[4] Xu SY, Sun K, Owusu-Ansah KG, et al. Central pancreatectomy for pancreatic schwannoma: A case report and literature review. World J Gastroentero. 2016;22(37):8439–8446. 10.3748/wjg.v22.i37.8439. 10.3748/wjg.v22.i37.8439 PMC5055874 27729750

[5] Sahin TK, Rizzo A, Aksoy S, et al. Prognostic significance of the Royal Marsden Hospital (RMH) score in patients with Cancer: a systematic review and Meta-analysis. Cancers (Basel). 2024;16(10). 10.3390/cancers16101835. 10.3390/cancers16101835 PMC11120545 38791914

[6] Tofigh AM, Hashemi M, Honar BN, et al. Rare presentation of pancreatic schwannoma: a case report. J Med Case Rep. 2008;2:268. 10.1186/1752-1947-2-268. 18694526 10.1186/1752-1947-2-268 PMC2526090

[7] Hirabayashi K, Yasuda M, Umemura S, et al. Cytological features of the cystic fluid of pancreatic schwannoma with cystic degeneration. A case report. Jop. 2008;9(2):203–8. 18326930

[8] Das Gupta TK, Brasfield RD. Tumors of peripheral nerve origin: benign and malignant solitary schwannomas. Ca-Cancer J Clin. 1970;20(4):228–33. 10.3322/canjclin.20.4.228. 10.3322/canjclin.20.4.228 4316984

[9] Zhang X, Siegelman ES, Lee MK, et al. Pancreatic schwannoma, an extremely rare and challenging entity: report of two cases and review of literature. Pancreatology. 2019;19(5):729–37. 10.1016/j.pan.2019.05.460. 31153779 10.1016/j.pan.2019.05.460

[10] Veron Sanchez A, Santamaria Guinea N, Cayon Somacarrera S, et al. Rare solid pancreatic lesions on cross-sectional imaging. Diagnostics (Basel). 2023;13(16). 10.3390/diagnostics13162719. 10.3390/diagnostics13162719 PMC10453474 37627978

[11] Varshney VK, Yadav T, Elhence P, et al. Preoperative diagnosis of pancreatic schwannoma - Myth or reality. J CANCER RES THER. 2020;16(Supple):S222–S226. 10.4103/jcrt.JCRT_730_18. 33380683 10.4103/jcrt.JCRT_730_18

[12] Fukuhara S, Fukuda S, Tazawa H, et al. A case of pancreatic schwannoma showing increased FDG uptake on PET/CT. Int J Surg Case Rep. 2017;36:161–166. 10.1016/j.ijscr.2017.05.031. 28599230 10.1016/j.ijscr.2017.05.031 PMC5466559

[13] Lugo-Fagundo E, Lugo-Fagundo C, Weisberg EM, et al. CT of pancreatic schwannoma. Radiol Case Rep. 2023;18 (5):2043–2046. 10.1016/j.radcr.2023.02.054. 37006832 10.1016/j.radcr.2023.02.054 PMC10050463

[14] Zhuo Y, Zhou X, Cao P et al. A rare case of benign pancreatic schwannoma with regional lymph node metastasis. Asian J Surg. 2023;46. 10.1016/j.asjsur.2023.04.085. 10.1016/j.asjsur.2023.04.085 37130777

[15] Shi Z, Cao D, Zhuang Q, et al. MR imaging features of pancreatic schwannoma: a Chinese case series and a systematic review of 25 cases. Cancer Imaging. 2021;21(1):23. 10.1186/s40644-021-00390-x. 33588954 10.1186/s40644-021-00390-x PMC7885599

[16] van Huijgevoort NCM, Del Chiaro M, Wolfgang CL et al. Diagnosis and management of pancreatic cystic neoplasms: current evidence and guidelines. NAT REV GASTRO HEPAT. 2019; 16. 10.1038/s41575-019-0195-x 10.1038/s41575-019-0195-x 31527862

[17] Papavramidis T, Papavramidis S. Solid pseudopapillary tumors of the pancreas: review of 718 patients reported in English literature. J AM COLL Surg. 2005. 10.1016/j.jamcollsurg.2005.02.011. 200 J AM COLL SURGEONS. 15922212 10.1016/j.jamcollsurg.2005.02.011

[18] Ghaneh P, Hanson R, Titman A, et al. PET-PANC: multicentre prospective diagnostic accuracy and health economic analysis study of the impact of combined modality 18fluorine-2-fluoro-2-deoxy-d-glucose positron emission tomography with computed tomography scanning in the diagnosis and management of pancreatic cancer. HEALTH TECHNOL ASSES. 2018;22. 10.3310/hta22070. 10.3310/hta22070 PMC5817411 29402376

[19] Vilmann P, Jacobsen GK, Henriksen FW, et al. Endoscopic ultrasonography with guided fine needle aspiration biopsy in pancreatic disease. GASTROINTEST ENDOSC. 1992;38(2):172–3. 10.1016/s0016-5107(92)70385-x. 1568614 10.1016/s0016-5107(92)70385-x

[20] Iemoto T, Sasaki A, Sanuki T et al. A case of pancreatic schwannoma with a focus on contrast-enhanced endoscopic ultrasonography. ENDOSCOPY. 2022; 54 ENDOSCOPY. 10.1055/a-1422-1763 10.1055/a-1422-1763 33910248

[21] Moussa S, Cruz S, Ingram M, et al. Peripancreatic schwannoma: a case report. Int J Surg Case Rep. 2021;83. 10.1016/j.ijscr.2021.105977. 10.1016/j.ijscr.2021.105977 PMC8164022 34022762

[22] Hanaoka T, Okuwaki K, Imaizumi H, et al. Pancreatic Schwannoma diagnosed by endoscopic ultrasound-guided fine-needle aspiration. Intern MED. 2021;60. 10.2169/internalmedicine.6129-20. 10.2169/internalmedicine.6129-20 PMC8170256 33250465

[23] Mikhetko A, Artemeva A, Ivko O, et al. CASE OF CYTOMORPHOLOGICAL DIAGNOSIS OF PANCREATIC SCHWANNOMA. Vopr Onkol. 2024;66. 10.37469/0507-3758-2020-66-3-296-301.

[24] Azami T, Takano Y, Niiya F, et al. A case of primary pancreatic schwannoma diagnosed by endoscopic ultrasound-fine needle aspiration. CLIN J GASTROENTEROL. 2020;13. 10.1007/s12328-020-01095-7. 10.1007/s12328-020-01095-7 31983049

[25] Yoshinaga S, Itoi T, Yamao K, et al. Safety and efficacy of endoscopic ultrasound-guided fine needle aspiration for pancreatic masses: a prospective multicenter study. DIGEST ENDOSC. 2020;32. 10.1111/den.13457. 10.1111/den.13457 31166046

[26] Hewitt MJ, McPhail MJ, Possamai L, et al. EUS-guided FNA for diagnosis of solid pancreatic neoplasms: a meta-analysis. GASTROINTEST ENDOSC. 2012;75(2):319–31. 10.1016/j.gie.2011.08.049. 22248600 10.1016/j.gie.2011.08.049

[27] Pecero-Hormigo MDC, Costo-Campoamor A, Cordero PG, et al. Pancreatic tail schwannoma. GASTROENT HEPAT-BARC. 2017;40. 10.1016/j.gastrohep.2016.06.005. 10.1016/j.gastrohep.2016.06.005 27496806

[28] Ercan M, Aziret M, Bal A, et al. Pancreatic schwannoma: a rare case and a brief literature review. Int J Surg Case Rep. 2016;22:101–4. 10.1016/j.ijscr.2016.03.014. 27084984 10.1016/j.ijscr.2016.03.014 PMC4844663

[29] Witkowski G, Kołos M, Nasierowska-Guttmejer A, et al. Neuroma (schwannoma). A rare pancreatic tumor. POL J SURG. 2019;92. 10.5604/01.3001.0012.8558. 10.5604/01.3001.0012.8558 32312928

[30] Ma Y, Shen B, Jia Y, et al. Pancreatic schwannoma: a case report and an updated 40-year review of the literature yielding 68 cases. BMC Cancer. 2017;17(1):853. 10.1186/s12885-017-3856-6. 29241452 10.1186/s12885-017-3856-6 PMC5731208

[31] Sung S, Rao R, Sharaiha RZ, et al. Fine-needle aspiration cytology of pancreatic Schwannoma. DIAGN CYTOPATHOL. 2017;45. 10.1002/dc.23656. 10.1002/dc.23656 28217914

[32] Tan G, Vitellas K, Morrison C, et al. Cystic schwannoma of the pancreas. ANN DIAGN PATHOL. 2003;7(5):285–91. 10.1016/s1092-9134(03)00082-0. 14571430 10.1016/s1092-9134(03)00082-0

[33] Wang S, Xing C, Wu H, et al. Pancreatic schwannoma mimicking pancreatic cystadenoma: a case report and literature review of the imaging features. Medicine. 2019;98(MEDICINE). 10.1097/MD.0000000000016095. 10.1097/MD.0000000000016095 PMC6587594 31192973

[34] Duma N, Ramirez DC, Young G, et al. Enlarging pancreatic Schwannoma: a Case Report and Review of the literature. Clin PRACT. 2015;5. 10.4081/cp.2015.793. 10.4081/cp.2015.793 PMC4745593 26918099

[35] Chen W, Cai G. Endoscopic ultrasound-guided fine-needle aspiration biopsy of gastric schwannoma: Cytomorphologic features and diagnostic pitfalls. Diagn Cytopathol. 2019;47(11):1218–1222. 10.1002/dc.24289. 31343112 10.1002/dc.24289

[36] Weiss SW, Langloss JM, Enzinger FM. Value of S-100 protein in the diagnosis of soft tissue tumors with particular reference to benign and malignant Schwann cell tumors. Lab Invest. 1983;49(3):299–308. 6310227

[37] Wang H, Zhang BB, Wang SF, et al. Pancreatic schwannoma: imaging features and pathological findings. HEPATOB PANCREAT DIS. 2019;19(2):200–2. 10.1016/j.hbpd.2019.07.008. 10.1016/j.hbpd.2019.07.008 31378472

[38] Ohbatake Y, Makino I, Kitagawa H, et al. A case of pancreatic schwannoma - the features in imaging studies compared with its pathological findings: report of a case. CLIN J GASTROENTEROL. 2014;7. 10.1007/s12328-014-0480-8. 10.1007/s12328-014-0480-8 26183748

[39] Marangos PJ, Parma AM, Goodwin FK. Functional properties of neuronal and glial isoenzymes of brain enolase. J NEUROCHEM. 1978;31(3):727–32. 10.1111/j.1471-4159.1978.tb07847.x. 681951 10.1111/j.1471-4159.1978.tb07847.x

[40] Xu CM, Luo YL, Li S et al. Multifunctional neuron-specific enolase: its role in lung diseases. BIOSCIENCE REP. 2019; 39. 10.1042/BSR20192732 10.1042/BSR20192732 PMC6859115 31642468

[41] Haimoto H, Takahashi Y, Koshikawa T et al. Immunohistochemical localization of gamma-enolase in normal human tissues other than nervous and neuroendocrine tissues. LAB INVEST. 1985; 52 (3): 257 – 63. PMID: 3974199. 3974199

[42] Theodosopoulos PV, Pensak ML. Contemporary management of acoustic neuromas. LARYNGOSCOPE. 2011;121(6):1133–7. 10.1002/lary.21799. 21557246 10.1002/lary.21799

[43] Birk H, Zygourakis CC, Kliot M. Developing an algorithm for cost-effective, clinically judicious management of peripheral nerve tumors. Surg Neurol Int. 2016; 7 Surg Neurol Int. 10.4103/2152-7806.189299 10.4103/2152-7806.189299 PMC5009575 27625890

[44] Holst JJ, Holland W, Gromada J et al. Insulin and Glucagon: Partners for Life. ENDOCRINOLOGY. 2017; 158. 10.1210/en.2016-1748 10.1210/en.2016-1748 PMC6061217 28323959

[45] Niwano F, Hiromine Y, Noso S, et al. Insulin deficiency with and without glucagon: a comparative study between total pancreatectomy and type 1 diabetes. J DIABETES INVEST. 2018. 10.1111/jdi.12799. 9 J DIABETES INVEST. 10.1111/jdi.12799 PMC6123030 29288524

[46] You DD, Choi SH, Choi DW, et al. Long-term effects of pancreaticoduodenectomy on glucose metabolism. ANZ J SURG. 2012;82(6):447–51. 10.1111/j.1445-2197.2012.06080.x. 22571457 10.1111/j.1445-2197.2012.06080.x

[47] Niwano F, Babaya N, Hiromine Y, et al. Glucose metabolism after pancreatectomy: Opposite extremes between Pancreaticoduodenectomy and Distal Pancreatectomy. J CLIN ENDOCR METAB. 2021;106(5):e2203–14. 10.1210/clinem/dgab036. 33484558 10.1210/clinem/dgab036 PMC8063252

[48] Müller MW, Friess H, Kleeff J et al. Middle segmental pancreatic resection: An option to treat benign pancreatic body lesions. ANN SURG. 2006; 244. 10.1097/01.sla.0000247970.43080.23 10.1097/01.sla.0000247970.43080.23 PMC1856616 17122616

[49] Han Y, Jang J, Kang J, et al. Endocrine function impairment after distal pancreatectomy: incidence and related factors. HPB. 2024;18. 10.1016/j.hpb.2016.03.106. 10.1007/s00268-015-3228-9 26330237

[50] Shirakawa S, Matsumoto I, Toyama H, et al. Pancreatic volumetric assessment as a predictor of new-onset diabetes following distal pancreatectomy. J GASTROINTEST SURG. 2012;16. 10.1007/s11605-012-2039-7. 10.1007/s11605-012-2039-7 PMC3508270 23054900

[51] Wu L, Nahm CB, Jamieson NB, et al. Risk factors for development of diabetes mellitus (type 3c) after partial pancreatectomy: a systematic review. CLIN ENDOCRINOL. 2020;92. 10.1111/cen.14168. 10.1111/cen.14168 32017157

[52] Bi S, Liu Y, Dai W, et al. Effectiveness and safety of central pancreatectomy in benign or low-grade malignant pancreatic body lesions: a systematic review and meta-analysis. Int J Surg. 2023;109(7):2025–2036. 10.1097/JS9.0000000000000326. 10.1097/JS9.0000000000000326 PMC10389642 37300889

[53] DiNorcia J, Ahmed L, Lee MK, et al. Better preservation of endocrine function after central versus distal pancreatectomy for mid-gland lesions. SURGERY. 2010;148. 10.1016/j.surg.2010.09.003. 10.1016/j.surg.2010.09.003 21134558

[54] Chen YW, Xu J, Li X, et al. Central pancreatectomy for benign or low-grade malignant pancreatic tumors in the neck and body of the pancreas. World J Gastrointest Surg. 2022;14. 10.4240/wjgs.v14.i9.896. 10.4240/wjgs.v14.i9.896 PMC9521472 36185570

[55] Ambiru S, Kato A, Kimura F, et al. Poor postoperative blood glucose control increases surgical site infections after surgery for hepato-biliary-pancreatic cancer: a prospective study in a high-volume institute in Japan. J HOSP INFECT. 2008;68. 10.1016/j.jhin.2007.12.002. 10.1016/j.jhin.2007.12.002 18294725

[56] Liao Y, Zhou W, Dai M, et al. Analysis of the clinical efficacy of laparoscopic middle pancreatectomy in the treatment of benign or low-grade malignant tumors of the pancreas. Front Oncol. 2023;13. 10.3389/fonc.2023.1231647. 10.3389/fonc.2023.1231647 PMC10652557 38023120

