# Supplementary figures and images for: Clinical benefits of central pancreatectomy for a patient with pancreatic schwannoma and diabetes
Source: World J Surg Oncol. 2025 Jan 3;23:2. doi: 10.1186/s12957-024-03646-5 (PMC11697483; doi:10.1186/s12957-024-03646-5)

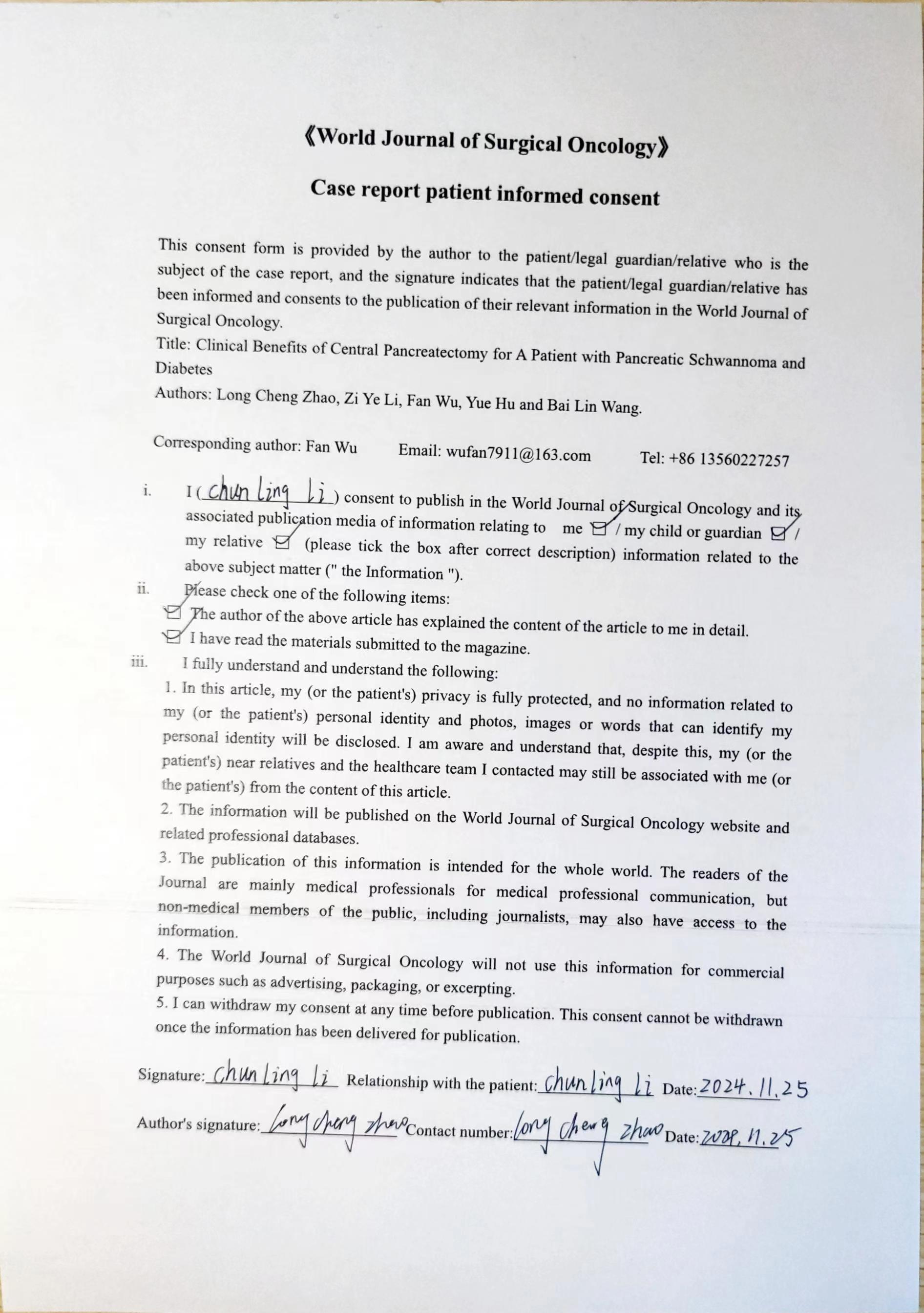

Supplement: Supplementary file 2 — Supplementary Material 2 [file 12957_2024_3646_MOESM2_ESM.jpg]
